# Supplementary material for: Mediterranean Diet, a Posteriori Dietary Patterns, Time-Related Meal Patterns and Adiposity: Results from a Cross-Sectional Study in University Students
Source: Diseases. 2022 Sep 11;10(3):64. doi: 10.3390/diseases10030064 (PMC9497476; doi:10.3390/diseases10030064)
Supplement: Supplementary file 1 [file diseases-10-00064-s001.zip › Supplementary Table S1.pdf]

Supplementary Table S1: Component loadings derived using Principal Component Analysis for the identification of dietary patterns.

	1	2	3	4	5	6
**Juices**	0.307	**0.810**	0.050	0.011	0.052	-0.047
**Sodas**	-0.161	**0.662**	0.062	0.156	0.044	0.153
**Liquid calories**	0.151	**0.954**	0.081	0.068	0.077	0.053
**Alcoholic drinks**	-0.068	0.103	0.003	0.190	**0.722**	0.132
**Total dairy**	**0.457**	0.168	0.122	0.213	**0.624**	0.048
**Vegetables**	**0.735**	-0.099	0.205	-0.063	0.036	0.066
**Legumes**	**0.481**	0.110	0.344	0.153	-0.071	-0.215
**Eggs**	0.232	0.024	0.110	0.297	**0.573**	-0.140
**Meats**	-0.024	0.177	0.062	**0.697**	0.256	0.323
**Poultry**	-0.071	0.159	0.059	**0.623**	0.176	0.374
**Fish and seafoods**	0.009	-0.004	0.026	**0.850**	0.046	-0.022
**Refined grains**	0.263	0.251	0.353	0.275	0.238	0.396
**Whole grains**	**0.538**	0.144	0.047	-0.017	0.369	0.051
**Fruits**	**0.765**	0.023	-0.031	-0.019	-0.082	0.047
**Other fats**	-0.088	0.106	**0.743**	-0.092	0.364	0.292
**Olive oil**	0.176	-0.006	**0.649**	0.208	-0.418	-0.136
**Total fats**	0.017	0.085	**0.951**	0.030	0.088	0.173
**Tea**	**0.466**	0.123	-0.152	-0.014	0.104	0.036
**Coffee**	-0.028	-0.140	-0.024	-0.212	0.351	0.296
**Nuts**	0.243	-0.210	-0.025	0.219	0.007	0.417
**Fast foods**	-0.049	0.099	0.050	0.188	-0.060	**0.765**
**Sweets (incl. honey/marmalade)**	0.060	0.233	0.299	0.040	0.219	**0.670**
**% Cumulative variance explained**	21.4	31.9	40.5	48.6	55.0	60.1

Higher absolute values of the loadings indicate that the meal is correlated with the respective component. Numbers in bold indicate absolute loadings greater than 0.45.

Pattern 1: legumes, vegetables, fruits, tea, dairy, whole grains
Pattern 2: Juices, sodas, liquid calories
Pattern 3: olive oil, fats
Pattern 4: meat, poultry, fish
Pattern 5: alcohol, eggs, total dairy
Pattern 6: fast foods, total sweets
