# Supplementary material for: Mediterranean Diet, a Posteriori Dietary Patterns, Time-Related Meal Patterns and Adiposity: Results from a Cross-Sectional Study in University Students
Source: Diseases. 2022 Sep 11;10(3):64. doi: 10.3390/diseases10030064 (PMC9497476; doi:10.3390/diseases10030064)
Supplement: Supplementary file 1 [file diseases-10-00064-s001.zip › Supplementary Table S2.pdf]

Supplementary Table S2: Component loadings derived using Principal Component Analysis for the identification of time- related meal patterns.

	Early eaters	*Medium eaters*	*Late eaters*
**Breakfast**	**0.726**	0.344	-0.341
**Snack**	**0.811**	-0.037	0.207
**Meal**	-0.007	**0.817**	-0.078
**Afternoon snack**	**0.720**	-0.063	0.346
**Dinner**	0.073	**0.672**	0.407
**Bedtime snack**	0.187	0.103	**0.869**
**% Cumulative variance**	33.7	53.07	70.0

Higher absolute values of the loadings indicate that the meal is correlated with the respective component. Numbers in bold indicate absolute loadings greater than 0.45.
